# Development and application of two novel monoclonal antibodies against overexpressed CD26 and integrin α3 in human pancreatic cancer

**DOI:** 10.1038/s41598-019-57287-w

**Published:** 2020-01-17

**Authors:** Gustavo A. Arias-Pinilla, Angus G. Dalgleish, Satvinder Mudan, Izhar Bagwan, Anthony J. Walker, Helmout Modjtahedi

**Affiliations:** 1 School of Life Sciences, Pharmacy and Chemistry, Kingston University London, Kingston-upon-Thames, Surrey UK; 2 Department of Cellular and Molecular Medicine, St George’s University of London, London, UK; 3 Department of Surgery, Imperial College London and The Royal Marsden Hospital, London, UK; 4 Department of Histopathology, Royal Surrey County Hospital, Guildford, UK

**Keywords:** Pancreatic cancer, Cancer

## Abstract

Monoclonal antibody (mAb) technology is an excellent tool for the discovery of overexpressed cell surface tumour antigens and the development of targeting agents. Here, we report the development of two novel mAbs against CFPAC-1 human pancreatic cancer cells. Using ELISA, flow cytometry, immunoprecipitation, mass spectrometry, Western blot and immunohistochemistry, we found that the target antigens recognised by the two novel mAbs KU44.22B and KU44.13A, are integrin α3 and CD26 respectively, with high levels of expression in human pancreatic and other cancer cell lines and human pancreatic cancer tissue microarrays. Treatment with naked anti-CD26 mAb KU44.13A did not have any effect on the growth and migration of cancer cells nor did it induce receptor downregulation. In contrast, treatment with anti-integrin α3 mAb KU44.22B inhibited growth *in vitro* of Capan-2 cells, increased migration of BxPC-3 and CFPAC-1 cells and induced antibody internalisation. Both novel mAbs are capable of detecting their target antigens by immunohistochemistry but not by Western blot. These antibodies are excellent tools for studying the role of integrin α3 and CD26 in the complex biology of pancreatic cancer, their prognostic and predictive values and the therapeutic potential of their humanised and/or conjugated versions in patients whose tumours overexpress integrin α3 or CD26.

## Introduction

Pancreatic cancer remains one of the deadliest cancer types. In 2018, there were an estimated 458,918 new cases of pancreatic cancer, and 432,242 deaths as a result of pancreatic cancer in 185 countries worldwide^1,2^. Pancreatic cancer is predicted to become the second leading cause of cancer death after lung cancer, within the next decade in Western countries^3^.

At present, the only curative treatment for patients with pancreatic cancer is surgery. However, only a minority of patients are eligible for resection and disease recurrence is a frequent event in many such patients. Historically, gemcitabine-based therapy has been the mainstay for treatment of pancreatic cancer^4^. More recently the combination of gemcitabine plus capecitabine has been regarded as the new standard of care in the adjuvant setting^5^. Patients with metastatic disease are treated with either FOLFIRINOX or gemcitabine plus nab-paclitaxel as first-line in patients with good performance status^6,7^.

In order to reduce the dismal pancreatic cancer mortality rates, it is essential to discover novel biomarkers for use in the early detection of pancreatic cancer, to discover novel therapeutic targets and to develop novel and more effective therapeutic agents^8,9^. Monoclonal antibodies (mAbs) are excellent tools for the discovery of novel overexpressed cell surface antigens and their specific targeting for diagnostic and therapeutic purposes^10,11^. To date, 36 mAbs have been approved for cancer treatment in the U.S. and/or European Union, although none for pancreatic cancer yet^12,13^. As tumour heterogeneity has been reported both between (i.e. inter-tumour heterogeneity) and within tumours (i.e. intra-tumour heterogeneity) in patients with pancreatic cancer, and between primary tumours and their metastatic counterparts, it has not been possible to find a magic and universal drug for the treatment of such patients^14,15^. As a result, over the past few years, our work has been focused on the discovery of overexpressed cell surface antigens in human pancreatic cancer using a panel of pancreatic cancer cell lines derived from patients at different stages of their disease as the source of tumour immunogen and in the antibody screening and the study of such mAbs for use in cancer diagnosis and therapy. We reported recently the development of two novel antibodies against an antigen with high level of expression in pancreatic cancer (i.e. CD109) using the human pancreatic cancer cell line BxPC-3 (derived from a primary tumour) as the source of tumour immunogen^9,16^. BxPC-3 is a moderate to poorly differentiated cell line derived from a 61-year-old Caucasian female with a primary body of pancreas adenocarcinoma in whom no metastatic disease was found and who died 6 months later despite chemotherapy and radiation^16^. Here, we report the development of two novel mouse mAbs using CFPAC-1, a cancer cell line established from liver metastasis of a patient with pancreatic cancer, as the source of tumour immunogen^17^. As there is no complete concordance between the expression level of some genes and their protein products in the primary pancreatic cancer and the corresponding metastatic lesions, our strategy was to develop other antibodies against antigens with high levels of expression in the primary and/or metastatic pancreatic cancer using both the primary and metastatic pancreatic cancer cell lines as immunogen^18–21^. Indeed, some of the immunogenic antigens may only be overexpressed in the primary tumour cells (i.e. BxPC-3) and not the metastatic pancreatic tumour cells and vice versa. Using a panel of human pancreatic cancer cell lines established from patients at different stages of their diseases and tumour tissue arrays from patients with pancreatic cancer, we found that the overexpressed target antigens recognised by these two novel antibodies are integrin α3 and CD26. Thus, in addition to their diagnostic and therapeutic potential, these antibodies will be valuable tools for unravelling the role of integrin α3 and CD26 in the progression and complex biology of pancreatic cancer

## Results

### Development of two novel mouse mAbs KU44.22B and KU44.13A

**Figure 1:**
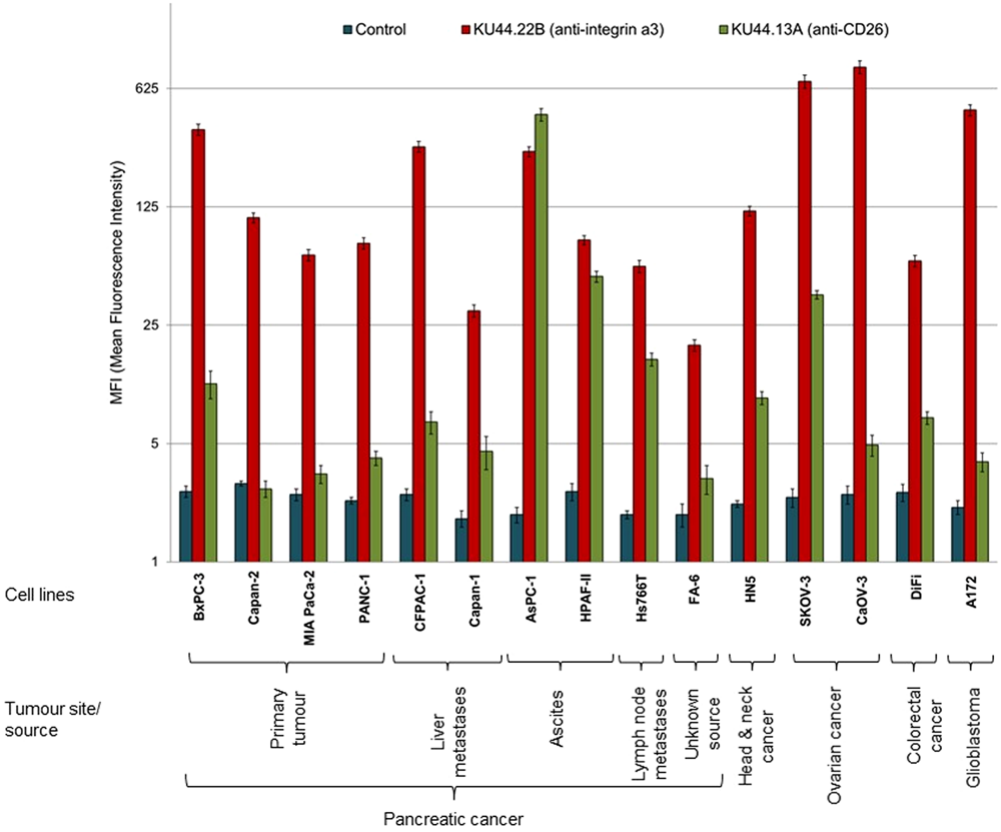
Expression level of the antigens recognised by novel mAbs KU44.22B and KU44.13A on human pancreatic and other cancer cell lines determined by flow cytometry.

A panel of hybridomas was generated by fusing lymphocytes from mice immunised with CFPAC-1 human pancreatic cancer cells and SP2 myeloma cells. Flow cytometry and ELISA revealed that the antigen recognised by mAb KU44.22B was widely expressed across pancreatic and other cancer cell lines, particularly in the human pancreatic cancer cells BxPC-3 (MFI = 357), CFPAC-1 (MFI = 283), AsPC-1 (MFI = 265) and Capan-2 (MFI = 107), as well as the ovarian cancer cell lines SKOV-3 (MFI = 687) and CaOV-3 (MFI = 836), the glioblastoma cell line A172 (MFI = 466) and the head and neck cancer cell line HN-5 (MFI = 118; Fig. 1 and Supplementary Figs. 1 and 2). On the other hand, mAb KU44.13A recognised an antigen with overexpression limited to AsPC-1 human pancreatic cancer cells (MFI = 437) and to a lower extent HPAF-II (MFI = 48) and the ovarian cell line SKOV-3 (MFI = 38; Fig. 1 and Supplementary Figs. 1 and 2). While in the ELISA, cancer cell monolayers were treated with the primary antibodies, for flow cytometry tumour cells were treated with trypsin prior to incubation with the primary antibody. As the results of ELISA show, the highest levels of target antigen recognised by mAb KU44.13A were found in human pancreatic cancer cell lines derived from ascites (i.e. AsPC-1 and HPAF-II) and lymph node metastasis (Supplementary Fig. 1). Using the mouse isotyping kits, novel mAbs KU44.22B and KU44.13A were found to be of IgG1κ and IgG2a isotypes respectively (data not shown).

### The two novel mAbs KU44.22B and KU44.13A are directed against integrin α3 and CD26 respectively

**Figure 2:**
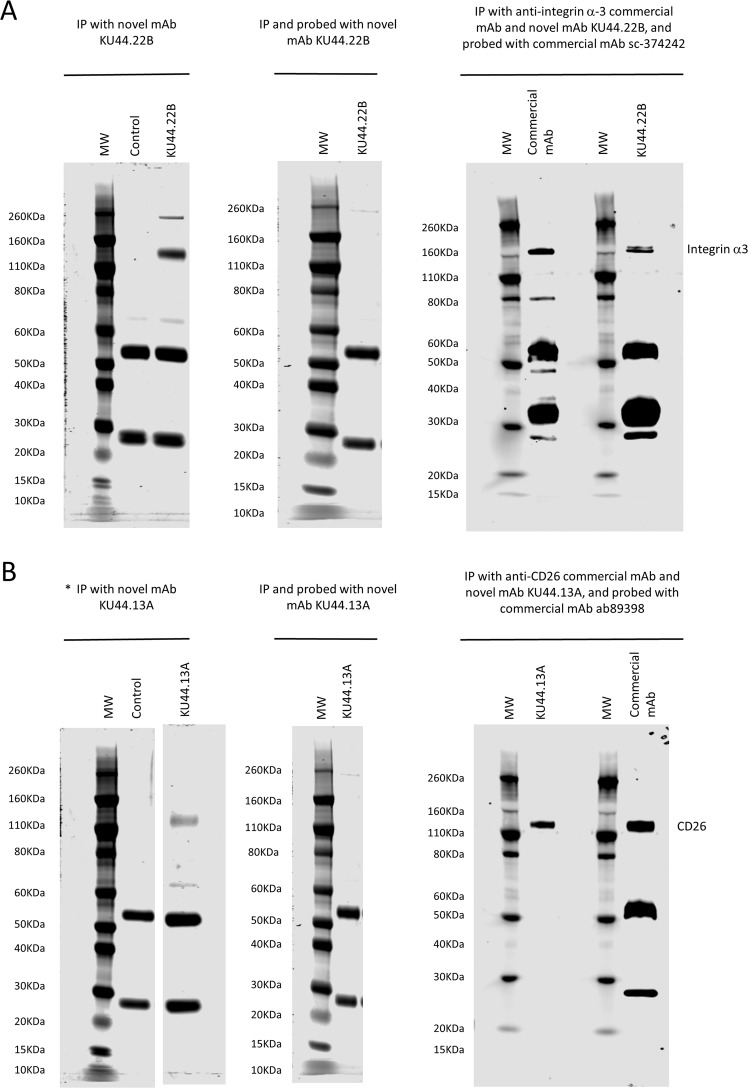
Immunoprecipitation and immunodetection by Western blot of (**A**) integrin α3 and (**B**) CD26 antigen with novel mAbs KU44.22B and KU44.13A using lysates from CaOV-3 ovarian cancer cells and AsPC-1 pancreatic cancer cells respectively. *Left panel:* Immunoprecipitation was performed with novel mAbs (**A**) KU44.22B and (**B**) KU44.13A (5 µg) using sheep anti-mouse dynabeads. Protein bands around ~140 KDa and ~ 260KDa were immunoprecipitated with mAb KU44.22B (A; left panel) and ~110 KDa by mAb KU44.13A (B; left panel) respectively and stained with SimplyBlue™ SafeStain. The ~50/25 KDa bands represent heavy and light chains of the anti-mouse antibody. *(**B**) left panel corresponds to a cropped gel; vertically sliced images of juxtaposed lanes that were non-adjacent in the gel have a clear separation delineating the boundary between the gels. *Middle panel:* Integrin α3 and CD26 antigen were immunoprecipitated with mAbs (**A**) KU44.22B and (**B**) KU44.13A (5 µg) respectively, and probed with the same antibody (30 µg/ml). Target antigens were not immunodetected with either of the mAbs. *Right panel:* Integrin α3 and CD26 antigen were immunoprecipitated with mAbs (**A**) KU44.22B and (**B**) KU44.13A respectively (5 µg) or commercial anti-integrin α3 and anti-CD26 antibodies (2 µg) and immunodetected with commercial mAbs sc-374242 and ab89398 as described in Methods. Immunodetection of target antigens immunoprecipitated by novel mAbs and probed with commercial mAbs confirmed the target identity. MW: molecular weight marker.

The results of SDS-PAGE stained gels showed that mAb KU44.22B immunoprecipitated a 140 KDa protein identified as integrin α3 by mass spectrometry whereas mAb KU44.13A immunoprecipitated a protein of approximately 110 kDa corresponding to CD26 from the lysates of CaOV3 and ASPC-1 cells respectively (Fig. 2A,B left panel, and Table 1, full-length gels are presented in Supplementary Fig. 3). In addition, using the lysis buffer described in methods, mAb KU44.22B precipitated a protein of approximately 260 kDa that could not be identified by mass spectrometry (Fig. 2A, left panel). However, using RIPA lysis buffer, this additional protein was not immunoprecipitated by mAb KU44.22B (Supplementary Fig. 3C). The faint protein band of ~65 KDa in the antibody samples and controls yielded a single peptide which matched to serum albumin. Neither of the two novel mAbs were able to detect the target antigens by Western blot (Fig. 2A,B, middle panel). To confirm the identity of the target antigens, immunoprecipitation with the novel mAbs and the anti-integrin α3 and anti-CD26 commercial antibodies, followed by immunodetection by Western blot with the same commercial antibodies was performed. As shown in Fig. 2A,B, right panel, both antigens were immunodetected with the commercial mAbs.

**Table 1:** Identification of proteins recognised by novel mAbs KU44.13A and KU44.22B by mass spectrometry.

Band No.	mAb	Protein Hits Matches/Sequences		
1	KU44.13A	P27487 Dipeptidyl peptidase 4/CD26 OS = Homo sapiens GN = DPP4 PE = 1 SV = 2 Mass: 88907 Score: 198 Matches: 5(5) Sequences: 3(3)		
		*Start-End*	*Score*	*Peptide*
		176–184	35	K.IEPNLPSYR.I
		598–611	89	R.LGTFEVEDQIEAAR.Q
		659–669	73	R.WEYYDSVYTER.Y
2	KU44.22B	P26006 Integrin alpha-3 OS = Homo sapiens GN = ITGA3 PE = 1 SV = 5 Mass: 117735 Score: 245 Matches: 4(4) Sequences: 4(4)		
		*Start-End*	*Score*	*Peptide*
		44–60	107	K.EAGNPGSLFGYSVALHR.Q
		68–76	46	R.YLLLAGAPR.E
		144–156	67	R.YTQVLWSGSEDQR.R
		462–471	27	R.ARPVINIVHK.T

### MAb KU44.22B inhibits the growth *in vitro* of Capan-2 cancer cells, increases migration of BxPC-3 and CFPAC-1 cancer cell lines and induces receptor downregulation and internalisation

**Figure 3:**
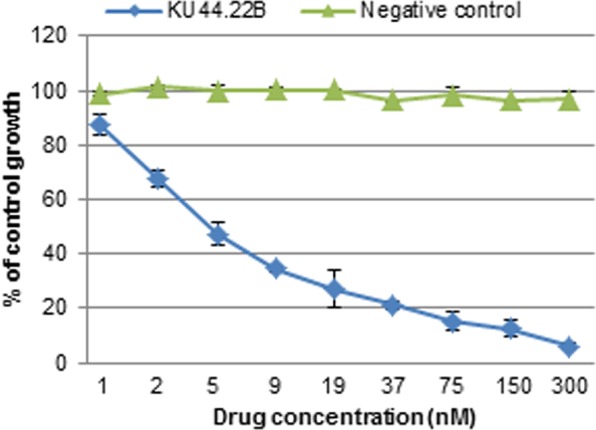
Effect of novel mAb KU44.22B on the growth of Capan-2 human pancreatic cancer cells determined by SRB assay as described in Methods. Novel mAb KU44.22B inhibits the growth of Capan-2 human pancreatic cancer cells with IC50 = 4.5 nM.

**Figure 4:**
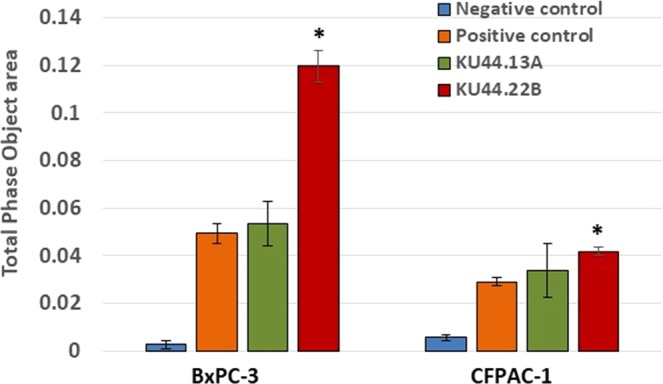
Effect of novel mAbs KU44.22B and KU44.13A on the migration of BxPC-3 and CFPAC-1 human pancreatic cancer cells using the IncuCyte ZOOM® Live-Cell Imaging instrument (Essen Bioscience, UK) as described in Methods. Treatment with mAb KU44.22B (300 nM) significatively increases the migration of BxPC-3 and CFPAC-1 cells.

**Figure 5:**
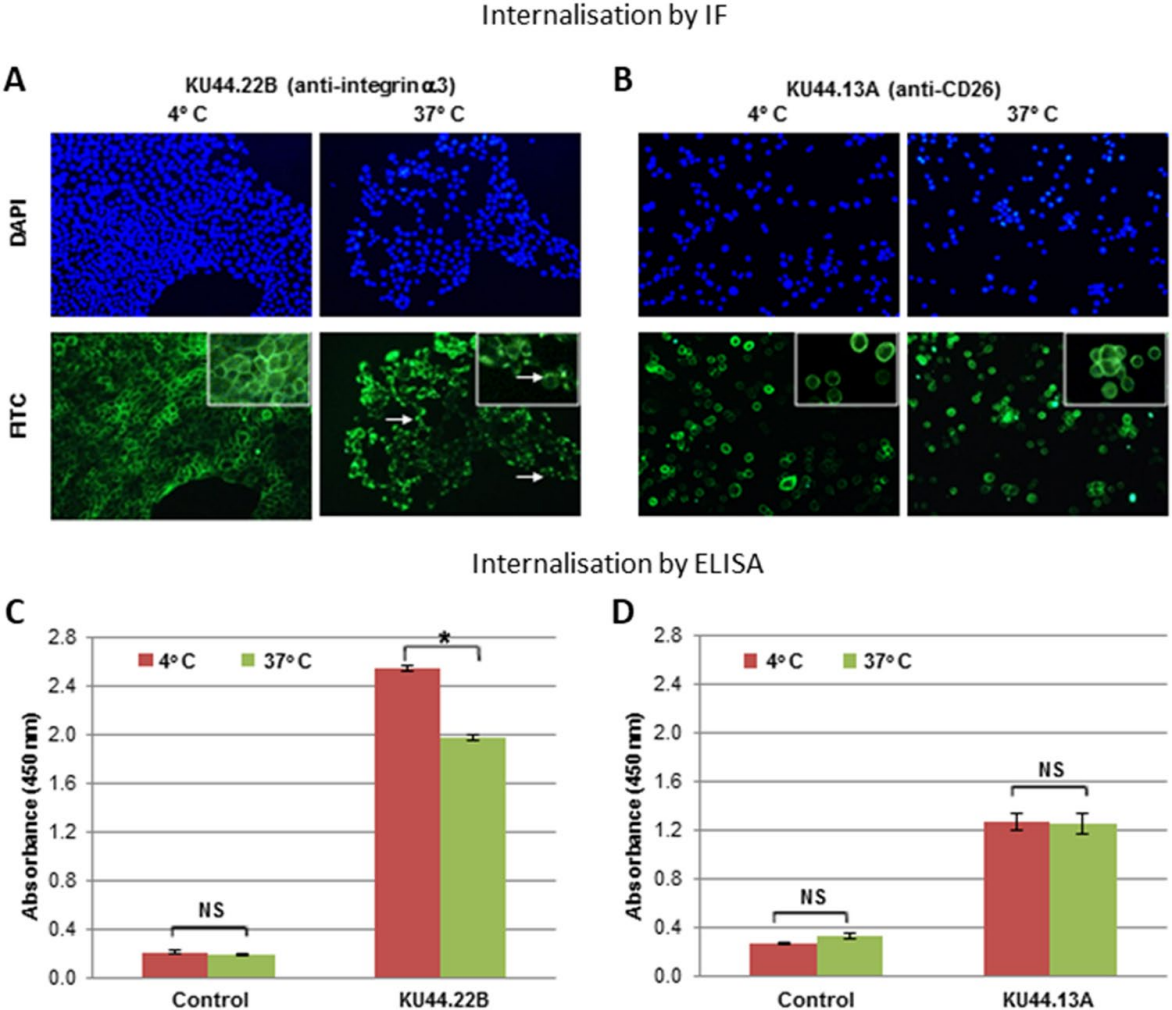
Internalisation studies of novel mAbs KU44.22B and KU44.13A in BxPC-3 and AsPC-1 human pancreatic cancer cells determined by (**A**,**B**) Immunofluorescence, BxPC-3 and AsPC-1 cancer cells were grown to near confluency and incubated with purified mAbs KU44.22B and KU44.13A respectively (50 μg/ml) or control (PBS/1% BSA) at 4 °C for 1 h and subsequently at 37 °C for extra 30 min to allow internalisation. Cells were then fixed, permeabilised and incubated with anti-mouse secondary antibody (Alexa Fluor 488; 1:200) at 4 °C for 1 h for detection using Nikon eclipse i80 microscope; and (**C**,**D**) ELISA, BxPC-3 and AsPC-1 cancer cells were grown to near confluency 96-well plates and incubated with purified mAbs KU44.22B and KU44.13A respectively (50 μg/ml) or control (PBS/1% BSA) at 4 °C for 1 h and subsequently at 37 °C for extra 30 min to allow internalisation. Cells were then fixed, permeabilised and incubated with HRP-conjugated rabbit anti-mouse (1:1000, STAR13B, AbD Serotec) and the absorbance of each sample measured at 450 nm.

We investigated the effect of treatment with these two novel antibodies on the growth and migration *in vitro* of a panel of human pancreatic and other cancer cell lines. At 300 nM, mAb KU44.22B inhibited the growth of Capan-2 human pancreatic cancer cells by 94% with an IC50 value of 4.5 nM (Fig. 3) whereas it inhibited the growth of CFPAC-1 cells by 20% (data not shown). Interestingly, treatment with this mAb did not have any effect on the growth of the other cell lines tested including the ovarian cancer cell lines SKOV-3 and CaOV-3, and the glioblastoma cell line A172, despite having higher levels of integrin α3 cell surface expression than Capan-2 cells (data not shown). On the other hand, treatment with mAb KU44.22B increased migration of BxPC-3 and to a lesser extent CFPAC-1 cancer cells (Fig. 4) and induced-receptor downregulation and internalisation (Fig. 5). In contrast, treatment with mAb KU44.13A did not have any effect on the growth or migration of any of the cell lines tested and did not induce receptor downregulation (data not shown, and Fig. 5).

### Immunohistochemical detection of the antigen recognised by novel mAbs KU44.22B and KU44.13A

**Figure 6:**
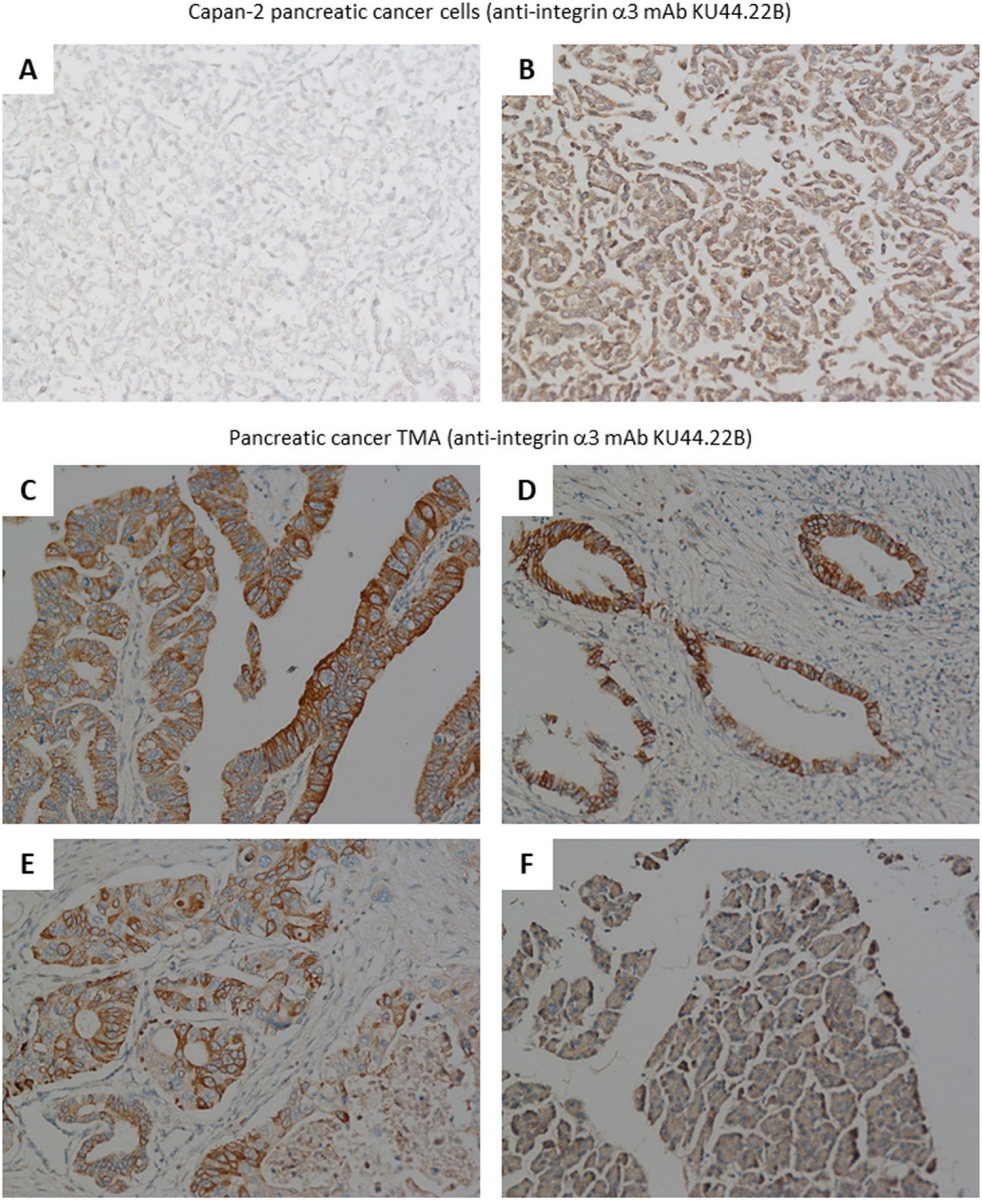
Examples of immunohistochemical staining of formalin-fixed, paraffin-embedded Capan-2 cancer cell pellets (**A-B**) and human pancreatic tissue microarrays (**C–F**) using novel mAb KU44.22B (15 μg/ml). Staining was performed as described in Methods section. (**A**) Negative control; (**B**) Staining of Capan-2 cells with mAb KU44.22B; (**C**) 2/3 + membrane, 2 + cytoplasm (C7); **(D**) 2 + membrane (B5); (**E**) 2 + membrane, 1 + cytoplasm (C3); (**F**) Normal pancreas tissue (F3); magnification 200×.

**Figure 7:**
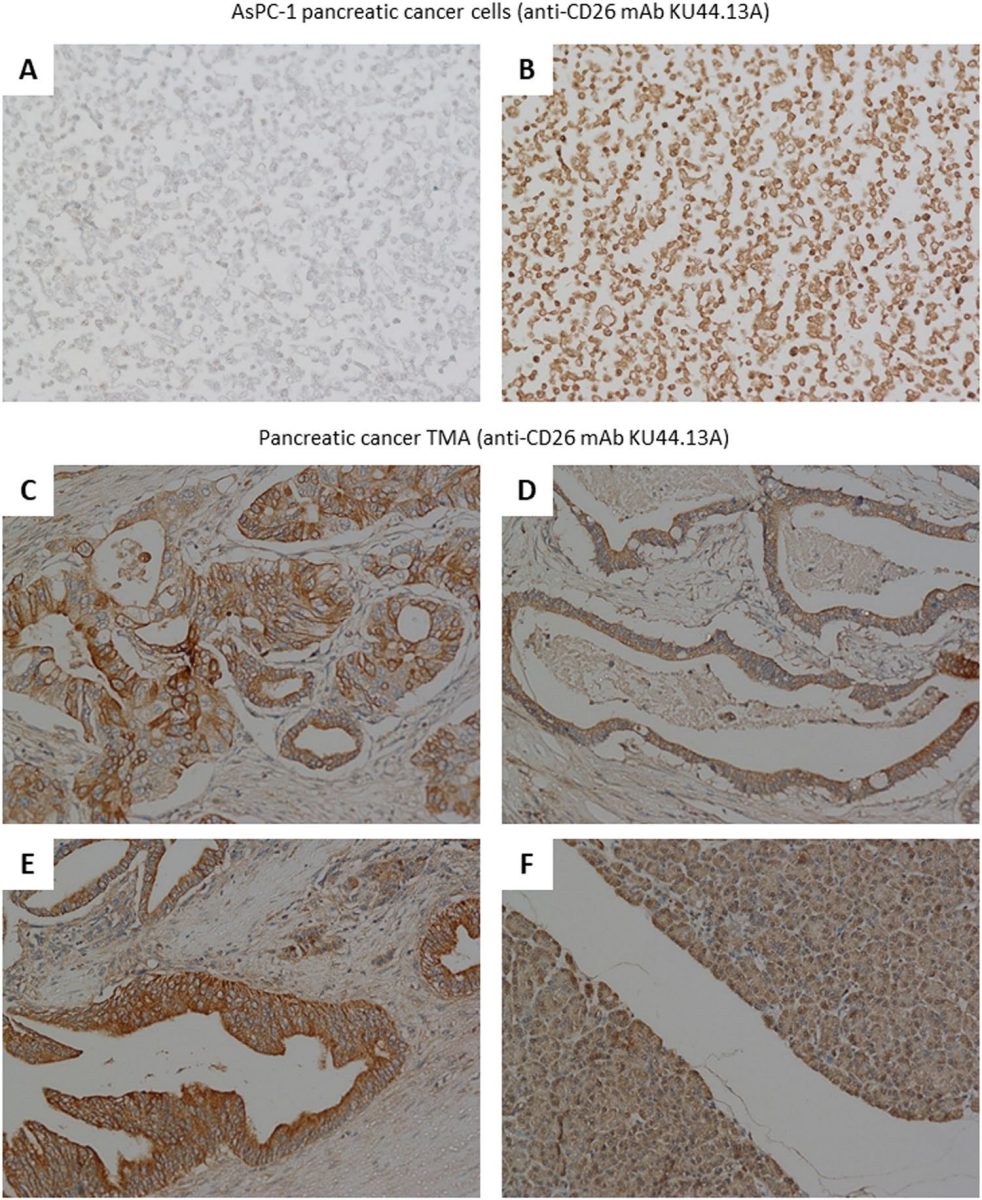
Examples of immunohistochemical staining of formalin-fixed, paraffin-embedded AsPC-1 cancer cell pellets (**A-B**) and human pancreatic tissue microarrays (**C–F**) using novel mAb KU44.13A (10 μg/ml). Staining was performed as described in Methods section. (**A**) Negative control; (**B)** Staining of AsPC-1 cells with mAb KU44.13A; (**C**) 2 + membrane, 1 + cytoplasm (C3); (**D**) 1 + membrane/cytoplasm (B3); (**E**) 2 + cytoplasm/membrane (D4); (**F**) Normal pancreas tissue (F8); magnification 200×.

To explore the diagnostic potential of the novel mAbs, immunohistochemical staining was performed in Capan-2 (mAb KU44.22B) and AsPC-1 (mAb KU44.13A) tumour cell pellets. The results showed that both mAbs were able to immunodetect the target antigens in formalin-fixed paraffin-embedded tumour sections (Figs. 6 and 7).

Then, we examined the relative expression of integrin α3 and CD26 (immunodetected by mAbs KU44.22B and KU44.13A respectively) in tissue arrays containing 48 specimens from patients with pancreatic cancer. Of these, three samples contained no tumour in the integrin α3 slide and 14 in the CD26 slide and therefore were excluded from the study. Of 45 cases, 64% of tissue samples were integrin α3 positive, with intensity ranging from 1 + weak (n = 17, 37.8%) to 2+ moderate (n = 12, 26.6%). Staining predominated in the membrane and/or the cytoplasm of cancer cells and did not correlate with the disease grade (Fig. 6C–E; Supplementary Table 1). In contrast, normal pancreatic tissue was negative or showed only weak cytoplasmic diffuse staining (Fig. 6F).

On the other hand, of 34 cases, CD26 was overexpressed in 64% of samples, with intensity 1 + weak (n = 16, 47.6%) to 2 + moderate (n = 6, 17.7%; Fig. 7C–E; Supplementary Table 2). Similarly, there was no correlation with tumour grading and weak cytoplasmic staining was seen in normal pancreatic tissue (Fig. 7F).

## Discussion

Pancreatic cancer is a highly aggressive and devastating cancer type, responsible for an increasing number of cancer related incidence and deaths^1,2,22^. The complex biology of pancreatic cancer and its microenvironment, the unavailability of reliable biomarkers for its early detection, the non-specific clinical presentation and the primary and secondary resistance to current therapeutic interventions are some of the attributing factors. Therefore, to improve the poor outcomes for patients diagnosed with pancreatic cancer, it is imperative to develop a screening technique for its early diagnosis, to discover novel therapeutic targets and to develop more effective and less toxic therapeutic agents. Monoclonal antibodies are excellent agents for use in both diagnosis and therapy of human cancer. The exquisite specificity of the antibodies for the target antigen also makes them ideal tools for investigating the biological, diagnostic, prognostic and predictive value of their target antigens and for use in targeted therapy of human cancers^10,11,23,24^. We have previously reported the generation of two novel anti-CD109 mAbs by means of hybridoma technology using BxPC-3 human pancreatic cancer cells, a cell line derived from a primary tumour, as the source of immunogen^9^. However, human cancers are heterogeneous in nature, a characteristic feature which contributes to the response of short duration or development of resistance to current therapies^21^. Both inter-tumour heterogeneity (i.e. heterogeneity between individuals), and intra-tumour heterogeneity (i.e. heterogeneity within the same tumour) as well as heterogeneity between the primary tumour and its metastatic counterparts have been reported in pancreatic cancer^14,15,19^. Here, using the human pancreatic cancer cell line CFPAC-1, which was isolated from a patient with liver metastasis, we describe the production and characterisation of two other novel mAbs. We have shown that these two novel antibodies are directed against integrin α3 (KU44.22B) and CD26 (KU44.13A).

We found overexpression of the target antigen integrin α3 recognised by the novel mAb KU44.22B in several human pancreatic cancer cell lines as well as human glioblastoma, ovarian and head and neck cancer cell lines as determined by ELISA and flow cytometry (Fig. 1 and Supplementary Figs. 1 and 2). This mAb inhibited the growth *in vitro* of Capan-2 cells (Fig. 3), increased migration of BxPC-3 and CFPAC-1 cells (Fig. 4) and induced receptor downregulation and internalisation (Fig. 5). While mAb KU44.22B was not able to recognise integrin α3 by Western blot, it proved useful for the immunohistochemical detection of the target antigen in formalin-fixed paraffin-embedded tissue sections from cancer cell pellets and tissue microarrays (Figs. 2 and 6).

Integrins are a family of heterodimeric (αβ) cell surface receptors that participate in cell-cell and cell-matrix interactions^25^. Their 18 α and 8 β subunits associate in different combinations and form at least 24 heterodimers with functional and tissue specificity^26^. Of these, integrin α3 (also known as ITGA3, CD49c, and VLA-3 subunit alpha) is a 140 KDa protein thought to be involved in the hepatocyte growth factor (HGF)/c-Met signal pathway contributing to tumour progression^27^. The integrin α3 subunit is believed to join a β1 subunit to form a complex that interacts with extracellular matrix proteins including members of the laminin family^28^. To our knowledge, this is the first report of anti-tumour activity *in vitro* of an anti-integrin α3 antibody in pancreatic cancer. At 300 nM, mAb KU44.22B inhibited the growth of Capan-2 cells by 94% and with an IC50 value of 4.5 nM (Fig. 3). However, as our hybridomas were grown in medium containing 3% FBS. it may be possible that the mouse antibodies purified on Protein G affinity column contain low level of calf antibodies. If this is the case, the actual IC50 value of mAb KU44.22B in Capan-2 cells could be even lower than 4.5 nM. The adaptation of the mouse hybridoma grown in serum-free medium or the purification of the mouse hybridoma supernatants using anti-mouse IgG affinity column could help increase the purity of the antibodies by eliminating potential contaminant calf antibodies. In another study, a mouse mAb (BCMab1), which was raised against aberrantly glycosylated integrin α3β1 on the human bladder cancer cell line T24, was accompanied by potent antitumor activity in subcutaneous and orthotopic bladder cancer mouse models^29^. Interestingly, we found that the anti-tumour activity of mAb KU44.22B was limited only to Capan-2 cells despite the high level of expression of its target antigen in other cancer cell lines (Fig. 1), which suggests that Capan-2 cell line is more dependent on integrin α3 for survival than any of the other cell lines examined. Further studies should be conducted to unravel the biological mechanisms that determine response to treatment with anti-integrin α3 mAb KU44.22B and to develop and investigate the therapeutic potential of the humanised version of this antibody in tumours with high levels of anti-integrin α3 *in vivo*.

Since Capan-2 is a non-migratory pancreatic cell line, the effect mAb KU44.22B on its migration could not be explored. However, treatment of BxPC-3 and CFPAC-1 cells with KU44.22B was accompanied by an increase in the migration of these tumour cells *in vitro* (Fig. 4). Indeed, integrin α3 has been reported to have both pro-migratory and anti-migratory effects in different tumour types. For example, depletion of integrin α3 was reported to increase the migration of prostate cancer cell lines and has been correlated with the disease grade and stage in prostate cancer^30^. Similarly, low integrin α3 expression was found to be associated with increased metastasis of certain colon cancers^31^. In contrast, in other studies integrin α3 was reported to have opposite effect by being overexpressed in glioma stem-like cells and contributing to the invasive nature of such cells^32,33^. ITGA3 (the protein coding gene for integrin α3) was found to be significantly associated with higher risk of recurrence and lower DFS in patients with colorectal tumours^34^ and shorter survival in pancreatic cancer^35^. High levels of ITGA3 correlate with more aggressive phenotypes and poor prognosis in patients with colorectal cancer and pancreatic ductal adenocarcinoma^36,27^. More recently, in another study overexpression of ITGA3 has been associated with a poor prognosis in patients with pancreatic cancer and ablation of ITGA3 was reported to be accompanied by a significant decrease in the EGFR expression and tumour growth^37^. ITGA3 expression may therefore be a biomarker of diagnostic and prognostic values in pancreatic cancer^38^. Our study here demonstrated integrin α3 overexpression in a wide TMA from pancreatic cancer patients by IHC with weak staining of normal pancreatic tissue (Fig. 6). Further studies with mAb KU44.22B are warranted and should help to determine the expression pattern, prognostic significance and predictive value of integrin α3 in patients with pancreatic cancer and other forms of cancer. This antibody is also an excellent tool in helping us understand the complex role of integrin α3 in tumour biology and its potential as target for therapy with monoclonal antibody-based drugs and other forms of therapeutics^26^.

Using immunoprecipitation followed by mass spectrometry, we found that the antigen recognised by the second mAb KU44.13A is CD26. Of the cell lines examined, CD26 overexpression was found to be limited to those derived from ascites specifically AsPC-1 pancreatic cells and to a lower extent HPAF-II pancreatic cancer cells and SKOV-3 ovarian cancer cells (Fig. 1 and Supplementary Figs. 1 and 2). Treatment with anti-CD26 mAb KU44.13A did not have any effect on cell proliferation, migration or receptor downregulation on the cell lines tested (data not shown and Fig. 5). While this mAb was not able to detect CD26 by Western blot, it was capable of detecting CD26 by immunohistochemistry in formalin-fixed paraffin-embedded tissue sections (Figs. 2 and 7).

CD26 (also known as Dipeptidyl peptidase-4 [DPP4/DPPIV] or ADCP2) is a 110 KDa membrane-associated peptidase. It is expressed in endothelial cells, fibroblasts and lymphocytes, on the apical surfaces of epithelial and acinar cells, and in a soluble form in plasma^39^. It is believed to play a role in tumour development through its association with intracellular proteins and its effect seems to be dependent on the tumour type and its microenvironment^40^. While CD26 has been shown to have a tumour suppressor effect in melanoma, ovarian cancer, non-small cell lung cancer, prostate cancer, endometrial cancer, neuroblastoma and glioma cell lines, it has been reported to be a marker of tumour aggressiveness in T-anaplastic large cell lymphoma, T-leukaemia, malignant mesothelioma, colorectal cancer and Ewing sarcoma cell lines^40,41^. Furthermore, CD26 has been reported to have higher levels of expression in pancreatic cancer tissue than in normal tissue and knockdown of CD26 expression inhibited cell growth, migration, invasion and colony formation, increases cell apoptosis of pancreatic cancer cells *in vitro* and decreased tumour growth and liver metastasis *in vivo*^42^. CD26 has been reported to be secreted in the serum of patients with different cancer types and therefore it has been proposed as a potential tumour biomarker of diagnostic and prognostic value in a wide range of cancers including pancreatic cancer^40–42^. CD26 has also been suggested as a cancer stem cell marker in different types of cancer and a potential therapeutic target^43–45^.

Although our anti-CD26 novel mAb KU44.13A did not show any effect on cell proliferation or migration on the panel of cell lines evaluated in this study, treatment with other anti-CD26 monoclonal antibodies have shown to reduce tumour growth *in vitro* and *in vivo* and improve survival in malignant mesothelioma, renal cell carcinoma and anaplastic large cell T-cell lymphoma^46–49^. We found moderate membranous and/or cytoplasmic staining of human pancreatic cancer tissue microarrays and cell pellets with anti-CD26 mAb KU44.13A (Fig. 7). Although there is no published research on the immunohistochemical staining of CD26 in pancreatic cancer, it has been shown that stromal CD26 expression following preoperative chemoradiotherapy has significant association with tumour recurrence and poor prognosis in patients with rectal cancer^50^. A positive association was also reported between high CD26 expression and tumour stage, development of metastasis and poor outcome in patients with colorectal cancer^51^. Finally, the results of a recent phase 1 clinical trial of the humanised anti-CD26 mAb YS110 in patients with mesothelioma, renal cell carcinoma and urothelial carcinoma CD26-expressing tumours showed a favourable safety profile and encouraged disease stabilisation in a subset of patients^52^. Further studies with our novel mAb KU44.13A in a larger panel of patients’ tumour specimens are warranted and should unravel the relative expression, prognostic significance and predictive value of CD26 in patients with pancreatic cancer as well as other cancer types.

In conclusion, in this study, we reported the production of two novel monoclonal antibodies against integrin α3 and CD26 on human pancreatic cancer cells, using the liver metastasis pancreatic cancer cell line CFPAC-1 as immunogen. These antibodies would be excellent tools for investigating the diagnostic, prognostic and predictive value of such antigens in pancreatic cancer, and studying their roles in the complex biology of pancreatic cancer and other cancer types. Further investigation are warranted to determine the therapeutic potential of these mAbs, particularly the conjugated and/or humanised versions with more effector ADCC (antibody-dependent cell-mediated cytotoxicity) and/or CDC (complement-dependent cytotoxicity) functions, for use in antibody-based targeted therapy of tumours with overexpression of these antigens^53,54^.

## Methods

### Cancer cell lines and cell culture

A panel of ten human pancreatic cancer cell lines (BxPC-3 [RRID:CVCL_0186], Capan-2 [RRID:CVCL_0026], MIA PaCa-2 [RRID:CVCL_0428], PANC-1 [RRID:CVCL_0480], AsPC-1 [RRID:CVCL_0152], HPAF-II [RRID:CVCL_0313], CFPAC-1 [RRID:CVCL_1119], Capan-1, Hs766T [RRID:CVCL_0334] and FA-6) were used in this study and cultured as described previously^9,55^. SP2 myeloma cells (RRID:CVCL_2199) were purchased from European Collection of Cell Cultures (ECACC, UK). HN5 (head and neck cancer cells), SKOV-3 and CaOV-3 (ovarian cancer cells), DiFi (colorectal cancer cells), A172 (glioblastoma cells) and SP2 myeloma cells were grown in Dulbecco’s Modified Eagle’s Medium supplemented with 10% FBS and antibiotics. All culture media and additives were purchased from Sigma Aldrich, UK. MIA PaCa-2, PANC-1, and Capan-1 were authenticated by American Type Culture Collection (ATCC, UK).

### Generation and screening of novel monoclonal antibodies

The mice immunisation was performed at St George’s University of London, following ethical approval and under Home Office animal license as described previously^9^. All experiments were performed in accordance with relevant guidelines and regulations. A group of female BALB/c mice (aged 5–6 weeks) were immunised subcutaneously (s.c) at two sites and intraperitoneally (i.p.) with a total number of 10 million CFPAC-1 human pancreatic cancer cells per immunisation per mouse (3 sites; 100 μl per site). Immunisation was repeated 2 times every 2 weeks and the final injection was administered 3–4 days before collection of lymphocytes from the spleen of immunised mice. B-lymphocytes derived from the spleen of immunised mice were fused with SP2 myeloma cells by 50% polyethylene glycol (PEG; Sigma Aldrich). Cells were cultured in HAT medium (Sigma Aldrich) supplemented with 20% FBS, 10% Hybridoma Cloning Supplement (Santa Cruz Biotechnology, USA) and antibiotics.

Antibodies secreted from novel hybridomas were screened by ELISA as described previously^9^. Newly formed positive hybridomas were selected, cloned twice by limiting dilution technique, adapted to growth medium containing 3% FBS, grown in roller bottles and the hybridoma supernatants were harvested and purified for further studies as described below.

### Flow cytometry

The cell surface expression of target antigens recognised by novel mAbs was determined using flow cytometry as described previously^9,56^. Briefly, cells were trypsinised and approximately 1 × 10^6^ tumour cells were incubated by rotation for 1 h at 4 °C with novel mouse mAbs KU44.22B or KU44.13A (10 μg/ml) or control (i.e. PBS), followed by incubation with FITC-conjugated goat anti-mouse IgG secondary antibody (1:200; STAR9B, AbD Serotec; RRID:AB_321920) for 45 min at 4 °C. A minimum of 10,000 events were recorded by excitation with an argon laser at 488 nm and analysed using the FL-1 detector (FITC detector; 525 nm) of a BD FACScalibur flow cytometer using CellQuest Pro software (Becton-Dickinson Ltd, UK; RRID:SCR_014489).

### Isotyping and purification of novel monoclonal antibodies

Isotyping of novel mAbs was determined using a mouse mAb isotyping kit (AbD Serotec, UK) according to the manufacturer’s protocol and the antibodies were purified by affinity chromatography as described previously^9^. Briefly, novel mouse mAbs were purified by salt fractionation (solid ammonium sulphate ([NH_4_]_2_SO_4_; 45% of saturation − 270 g/L; Fisher Scientific) followed by affinity chromatography using a 5 ml HiTrap Protein G HP column in an ÄKTAprime plus chromatography system (GE Healthcare, UK), as described previously^9^. The purified antibodies were filtered through a 0.2 µm syringe filter (Merck Millipore, UK), aliquoted and stored at −20 °C for further studies.

### Internalisation studies

Immunofluorescence staining of tumour cells and ELISA were used to determine whether treatment with novel antibodies resulted in down-regulation of the target antigen as described previously^9^. Briefly, pancreatic cancer cells were grown to near confluency in RPMI/10% FBS in Lab-Tek 8-well chamber slides (VWR, UK) or 96-well plates, respectively. Cells were incubated with mAbs KU44.22B or KU44.13A (50 µg/ml) or control (i.e. PBS/1%BSA alone) for 1 h at 4 °C to allow antibody binding, followed by incubation at 37 °C for 30 min to allow antibody internalisation. A control slide was maintained at 4 °C. Cells were fixed with 4% formaldehyde for 10 min, the cell membrane permeabilised with 0.5% Triton-X 100 for 15 min and non-specific binding blocked with PBS/1%BSA for 1 h at 4 °C. Cells in immunofluorescence slides were then incubated with Alexa Fluor 488 secondary antibody (1:200; Fisher Scientific; RRID:AI_1001) for 1 h at 4 °C, mounted in Vectashield with DAPI (Vector laboratories, UK) and examined using Nikon eclipse i80 microscope and Nikon NIS-Elements software as described previously (RRID:SCR_014329)^57^. Cells in ELISA plates were incubated under the same conditions above with the primary antibodies and then incubated with HRP-conjugated rabbit anti-mouse (1:1000, STAR13B, AbD Serotec; RRID:AB_321921). Following several washes, the absorbance of each sample was measured at 450 nm as described previously^9^

### Effect of novel mAbs on growth and migration of pancreatic cancer cells

The effect of novel mAbs on the growth of human cancer cell lines was investigated using the Sulforhodamine B (SRB) colorimetric assay^9,58^. Gen5 software was used to determine the IC50 through non-linear least squares curve fitting, as described previously^56,59^.

The effect of novel mAbs on the migration of human pancreatic cancer cells BxPC-3, AsPC-1 and CFPAC-1 was investigated using the IncuCyte ZOOM® Live-Cell Imaging instrument (Essen Bioscience, UK) as described previously^9^. Approximately 1 × 10^3^ cancer cells per well were seeded in duplicate in an IncuCyte™ ClearView 96-well Cell Migration Plate (Essen Bioscience, UK) along with 300 nM mAbs or control (i.e. medium alone). Cells were allowed to settle for 15 min at room temperature before transfer to an incubator at 37 °C for 30 min to pre-incubate the cells in the presence of treatment. Then, 200 μl of chemoattractant (i.e. 10% FBS medium) or control (i.e. 0.5% FBS medium) were added to the appropriate wells of the reservoir plate and the insert plate placed into the pre-filled reservoir plate. The plate was then transferred to the IncuCyte ZOOM® Live-Cell Imaging Instrument (Essen Bioscience) and allowed to warm to 37 °C for 15 min before any condensation accumulated on the plate lid or bottom was wiped away. The plate was imaged at 10x objective using the Chemotaxis Scan Type - Phase channel. The IncuCyte™ Chemotaxis Cell Migration Software Module (Essen Bioscience) was used for data analysis. Whole-well images of cells on both the bottom and the top of the plate membrane were captured every 2 h over 48 h and all images were processed using automatic algorithms to quantify cell area on each side of the membrane.

### Immunoprecipitation and mass spectrometry

To identify the target antigens recognised by the novel antibodies, immunoprecipitation and mass spectrometry were performed using protein identification service provided by the University of York (UK) as described previously^9^. Briefly, novel mAbs (5 μg) were incubated overnight at 4 °C by gentle rotation (14 rpm) with 1 ml AsPC-1 (mAb KU44.13A) or CaOV-3 (mAb KU44.22B) tumour cell lysates (prepared with lysis buffer containing 50 mM Tris-HCl pH 7.2, 150 mM NaCl, 2 mM MgCl_2_, 2 mM CaCl_2_, 0.1% NaN_3_, 100 mM DTT, 1% Triton X-100 and 50 mM N-ethylmaleimide), and then incubated with 50 μl pre-washed Dynabeads sheep anti-mouse IgG for 1 h at 4 °C (ThermoFisher Scientific). The immunocomplexes were captured on a DynaMag™-2 for 2 min, the supernatants aspirated, and the samples washed 3 times with PBS. The complexes were then eluted by mixing beads with LDS sample buffer (25% NuPAGE LDS buffer [4 × ], 10% reducing agent [10 × ] and 65% distilled water; Invitrogen, UK), heated to 95 °C for 5 min and analysed by SDS-PAGE. The SDS-PAGE gels were stained with SimplyBlue™ SafeStain (ThermoFisher Scientific) and the protein bands were excised (Supplementary Fig. 3A,B), and in gel digested with trypsin for subsequent mass spectrometry analysis.

Identification of isolated protein was performed by mass spectrometry under contract at the University of York’s protein identification facility, as described previously^9^. The UniProt_human_SP database was used for protein identification.

### Western blotting

The ability of the novel antibodies to recognise the target antigen in Western blot was investigated as described previously^9^. Briefly, proteins immunoprecipitated with novel mAbs KU44.22B and KU44.13A (5 µg) or commercial mAbs anti-integrin α3 (2 µg; sc-374242; Santa Cruz Biotechnology, UK; RRID:AB_10985868) or anti-CD26 (2 µg; ab89398; Abcam, UK; RRID: AB_2277416) were analysed by SDS-PAGE under reducing conditions, prior to Western blotting. The transfer of proteins from 4–12% Bis-Tris-gels to Immobilon-FL PVDF membranes (Merck Millipore, UK) was performed using the XCell II^TM^ Mini-Cell Blot Module kit (Invitrogen, UK) at a constant voltage of 30 V on ice for 2 h. PVDF membranes were probed with novel mAbs (30 μg/ml), commercial anti-integrin α3 mouse mAb (1:100 dilution) or commercial anti-CD26 rat mAb (1:500 dilution) overnight at 4 °C and subsequently incubated for 1 h at room temperature with secondary goat anti-mouse antibody (1:10,000; RRID:926_32210) or secondary goat anti-rat antibody (1:5,000; RRID:925-68076, both from LI-COR Biosciences, UK) respectively. For visualisation, the blots were analysed using the Oddysey® CLx instrument (LI-COR Biosciences; RRID:SCR_014579).

### Immunohistochemical staining

AsPC-1 and Capan-2 cancer cell pellets and human pancreatic cancer tissue microarrays (PA483e, Insight Biotechnology/US Biomax) were used to determine whether novel monoclonal antibodies immunodetect the target antigens in formalin-fixed paraffin-embedded tissue sections using the VENTANA BenchMark ULTRA IHC/ISH System (Roche, UK). Tissue sections were deparaffinised and rehydrated through a series of alcohols followed by heat induced antigen retrieval with standard CC1 (Tris-EDTA buffer pH 7.8) at 95 °C for 36 min and incubation for 1 h with either mAb KU44.22B (15 µg/ml) or mAb KU44.13A (10 µg/ml). An anti-mouse IgG detection system (UltraView Universal DAB Detection Kit, Ventana) was used for amplification and primary antibody detection. The slides were then counterstained with haematoxylin II for 8 min followed by Bluing reagent. The slides were then washed, dehydrated, cleared, mounted in DPX mounting medium (VWR) and coverslipped as described previously^60^.

## Supplementary information

SupplementaryInformation

## Data Availability

All data generated or analysed during this study are included in this published article (and its Supplementary Information Files).
