# Supplementary material for: Development and application of two novel monoclonal antibodies against overexpressed CD26 and integrin α3 in human pancreatic cancer
Source: Sci Rep. 2020 Jan 17;10:537. doi: 10.1038/s41598-019-57287-w (PMC6969035; doi:10.1038/s41598-019-57287-w)
Supplement: Supplementary file 1 — SupplementaryInformation [file 41598_2019_57287_MOESM1_ESM.docx]

**Development and application of two novel monoclonal antibodies against overexpressed CD26 and integrin α3 in human pancreatic cancer**

Gustavo A Arias-Pinilla^1^, Angus G Dalgleish^2^, Satvinder Mudan^3^, Izhar Bagwan^4^, Anthony J Walker^1^, Helmout Modjtahedi^1^*.

**Supplementary Figure 1.** Expression level of the antigens recognised by novel mAbs KU44.22B and KU44.13A on human pancreatic cancer cell lines determined by ELISA. Results are presented as mean absorbance **±** SD.


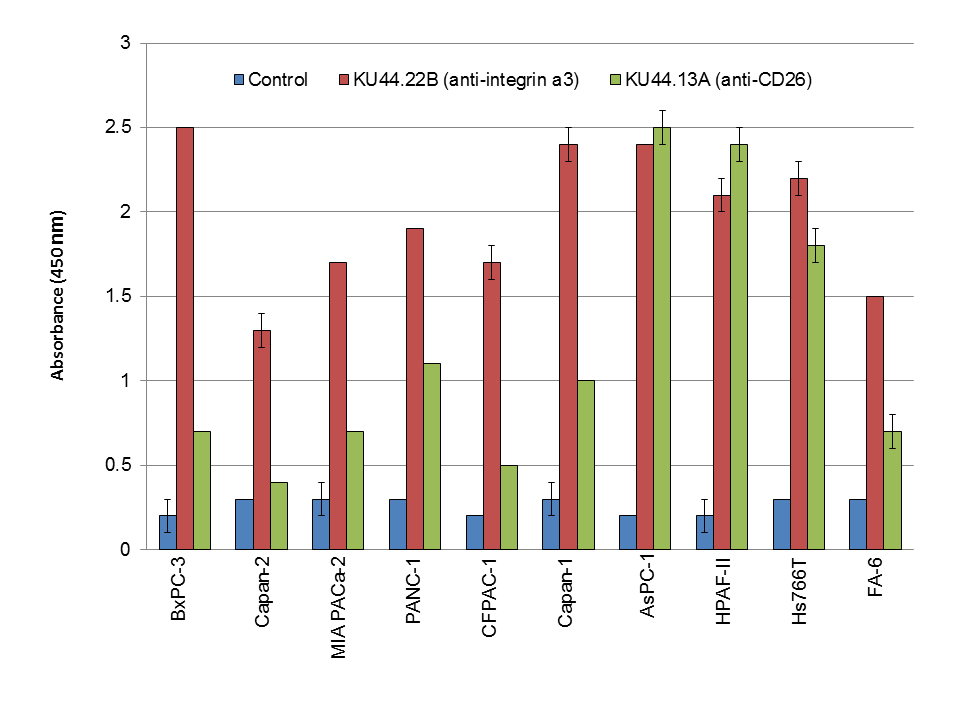


**Supplementary Figure 2.** Histograms of expression level of the antigens recognised by novel mAbs KU44.22B and KU44.13A on human pancreatic and other cancer cell lines determined by flow cytometry.


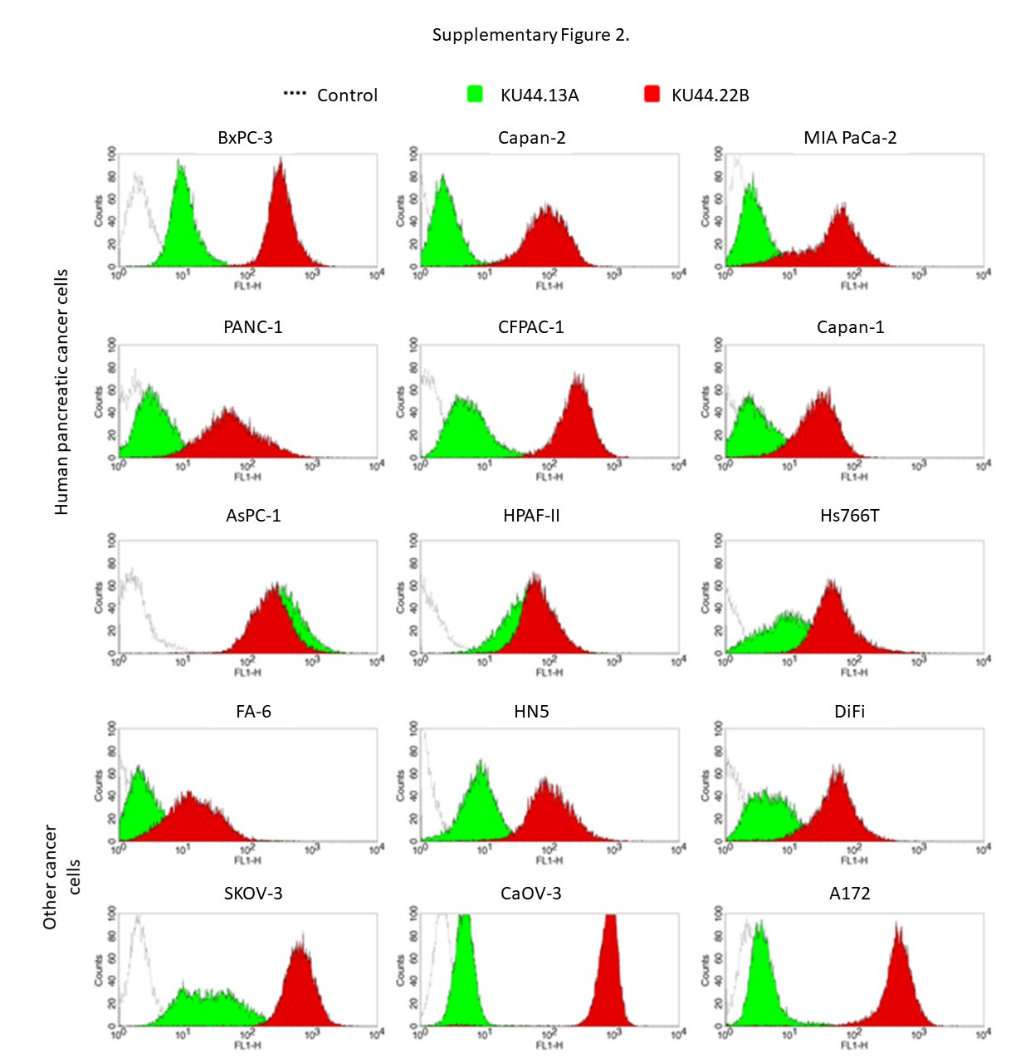


**Supplementary Figure 3.** Full-length gel of immunoprecipitation studies with novel mAbs KU44.22B and KU44.13A. A. Immunoprecipitation of CaoV3 tumour cell lysates with mAb KU4.22B. B. Immunoprecipitation of AsPC-1 tumour cell lysates with mAb KU44.13A SDS-PAGE gel was stained with SimplyBlue™ SafeStain and the protein bands highlighted were excised from these gels, in gel digested with trypsin and subject to mass spectrometry analysis as described in Methods section. C. Immunoprecipitation of CaoV3 tumour cell lysates with mAb KU4.22B using RIPA lysing buffer. Shaded areas on the whole gels corresponds to immunoprecipitation with other mAbs not relevant to this manuscript.

3A)

3B)

3C)


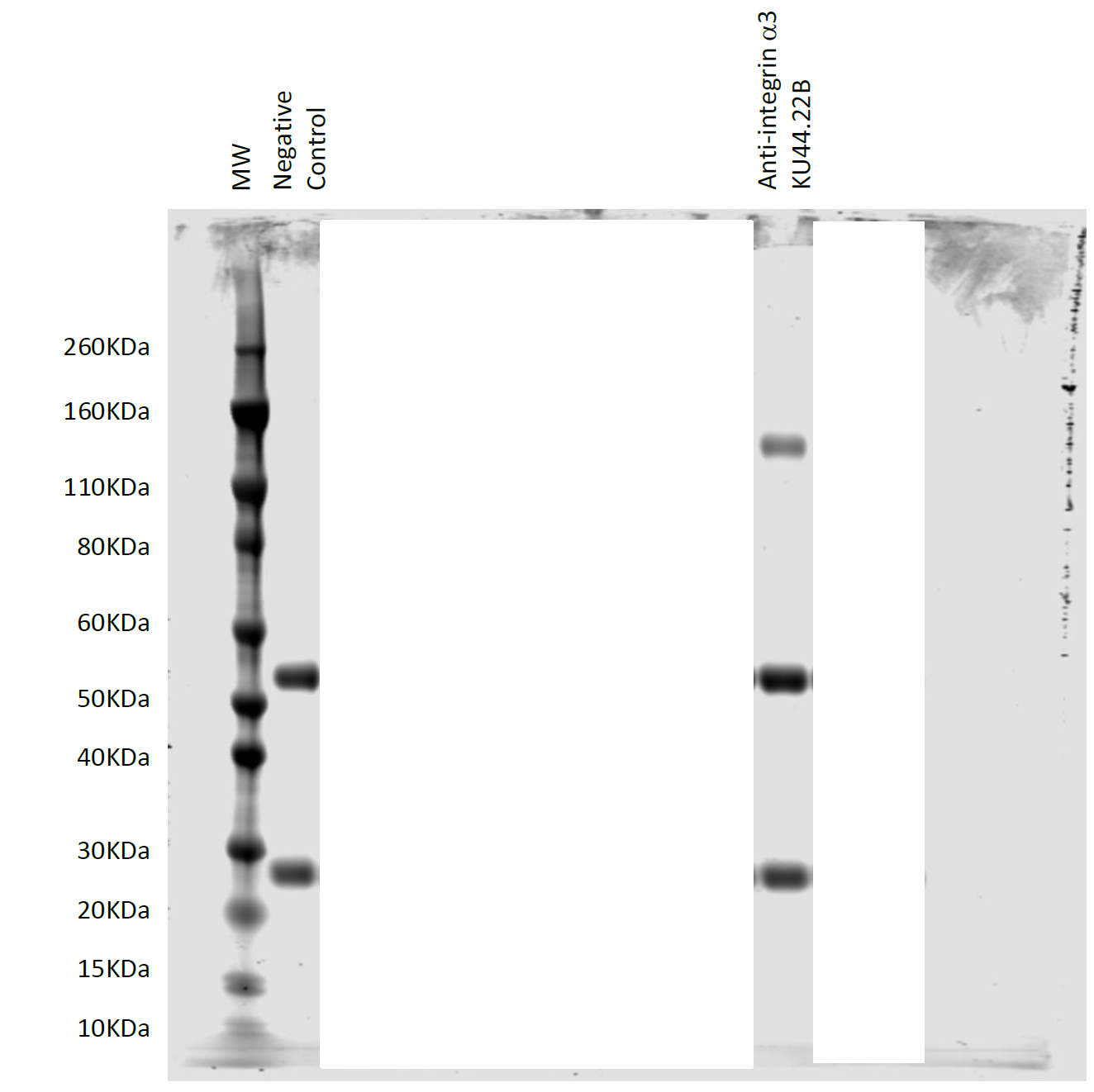

**Supplementary Table 2.** Patient characteristics, disease grade and stage, and staining intensity and distribution of pancreatic cancer samples in tissue microarray with anti-CD26 mAb KU44.13A (Cat No. PA483e, Biomax US).

| Position | Age | Sex | Pathology diagnosis | TNM | Grade | Stage | Type | Scoring IHC |
| --- | --- | --- | --- | --- | --- | --- | --- | --- |
| A1 | 72 | F | Duct adenocarcinoma | T3N0M0 | 1 | IIA | Malignant | no tumour |
| A2 | 58 | F | Duct adenocarcinoma (sparse) | T3N1M0 | 1 | IIB | Malignant | negative |
| A3 | 47 | M | Duct adenocarcinoma | T2N0M0 | 1 | IB | Malignant | no tumour |
| A4 | 67 | F | Duct adenocarcinoma | T3N1M0 | 1 | IIB | Malignant | no tumour |
| A5 | 34 | M | Duct adenocarcinoma | T3N0M0 | 1 | IIA | Malignant | no tumour |
| A6 | 56 | F | Duct adenocarcinoma | T2N0M0 | * | IB | Malignant | no tumour |
| A7 | 39 | M | Duct adenocarcinoma | T3N0M0 | 2 | IIA | Malignant | no tumour |
| A8 | 40 | M | Duct adenocarcinoma | T2N1M0 | 1 | IIB | Malignant | no tumour |
| B1 | 80 | M | Adenocarcinoma | T2N0M0 | 1 | IB | Malignant | negative |
| B2 | 51 | M | Duct adenocarcinoma | T3N1M0 | * | IIB | Malignant | negative |
| B3 | 56 | F | Duct adenocarcinoma | T2N0M0 | 1 | IB | Malignant | 1+ m/c |
| B4 | 66 | M | Duct adenocarcinoma with tumor embolus | T2N0M1 | 2 | IV | Malignant | 1+ m/c |
| B5 | 54 | M | Duct adenocarcinoma | T2N0M0 | 2 | IB | Malignant | no tumour |
| B6 | 56 | M | Duct adenocarcinoma | T2N1M0 | 1 | IIB | Malignant | no tumour |
| B7 | 58 | F | Duct adenocarcinoma | T2N1M0 | 1 | IIB | Malignant | no tumour |
| B8 | 44 | M | Duct adenocarcinoma | T3N0M0 | 2 | IIA | Malignant | no tumour |
| C1 | 65 | M | Adenocarcinoma | T3N0M0 | 2 | IIA | Malignant | negative |
| C2 | 66 | F | Adenocarcinoma invades the duodenal muscular layer | T2N0M0 | 2 | IB | Malignant | negative |
| C3 | 54 | M | Duct adenocarcinoma | T3N0M0 | 2 | IIA | Malignant | 2+ m/c |
| C4 | 77 | M | Duct adenocarcinoma | T1N0M0 | 2 | IA | Malignant | 1+ m/c |
| C5 | 42 | M | Duct adenocarcinoma | T3N0M0 | 2 | IIA | Malignant | 2+ m/c |
| C6 | 74 | M | Duct adenocarcinoma | T2N0M0 | 2 | IB | Malignant | negative |
| C7 | 46 | F | Papilary adenocarcinoma | T2N0M0 | 2 | IB | Malignant | no tumour |
| C8 | 68 | F | Duct adenocarcinoma | T2N0M0 | 2 | IB | Malignant | negative |
| D1 | 62 | F | Adenocarcinoma | T3N1M1 | 2 | IV | Malignant | negative |
| D2 | 72 | M | Adenocarcinoma | T3N0M0 | 2 | IIA | Malignant | negative |
| D3 | 45 | M | Adenocarcinoma | T3N0M0 | 2 | IIA | Malignant | 1+ m/c |
| D4 | 51 | F | Adenocarcinoma | T3N0M0 | 2 | IIA | Malignant | 2+ m/c |
| D5 | 49 | M | Adenocarcinoma | T3N1M0 | 2--3 | IIB | Malignant | 1+ m/c |
| D6 | 47 | F | Adenocarcinoma | T3N1M0 | 3 | IIB | Malignant | negative |
| D7 | 62 | F | Adenocarcinoma with squamous cell carcinoma differentiation | T3N0M0 | 3 | IIA | Malignant | negative |
| D8 | 56 | M | Adenocarcinoma | T2N0M0 | 3 | IB | Malignant | 1+ c |
| E1 | 66 | F | Mucinous adenocarcinoma | T2N1M0 | 3 | IIB | Malignant | negative |
| E2 | 60 | M | Adenocarcinoma invades nerve | T2N1M0 | 3 | IIB | Malignant | no tumour |
| E3 | 61 | F | Duct adenocarcinoma | T2N0M0 | 3 | IB | Malignant | 1+ m/c |
| E4 | 60 | M | Adenocarcinoma | T3N0M0 | 3 | IIA | Malignant | 1+ m/c |
| E5 | 55 | F | Adenocarcinoma | T2N0M0 | 3 | IB | Malignant | 1+ c |
| E6 | 52 | M | Adenocarcinoma | T2N0M0 | 3 | IB | Malignant | 1+ m/c |
| E7 | 40 | M | Acinic cell carcinoma | T1N0M0 | - | IA | Malignant | 1+ c |
| E8 | 62 | M | Squamous cell carcinoma | T3N0M0 | 3 | IIA | Malignant | 1+ c |
| F1 | 47 | M | Pancreas tissue | - | - | - | Normal | 1+ c |
| F2 | 45 | M | Pancreas tissue | - | - | - | Normal | 1+ c |
| F3 | 40 | M | Pancreas tissue | - | - | - | Normal | 2+ c islet |
| F4 | 40 | F | Pancreas tissue | - | - | - | Normal | 1+ c |
| F5 | 42 | F | Pancreas tissue | - | - | - | Normal | no tumour |
| F6 | 21 | F | Pancreas tissue | - | - | - | Normal | 2+ c |
| F7 | 35 | M | Pancreas tissue | - | - | - | Normal | 2+ c |
| F8 | 35 | M | Pancreas tissue | - | - | - | Normal | 1+ c |
